# Cryptococcal Lymphadenitis in an HIV-Infected Patient: A Rare Manifestation of Immune Reconstitution Inflammatory Syndrome

**DOI:** 10.3390/diseases6030070

**Published:** 2018-07-28

**Authors:** Jose Armando Gonzales Zamora, Yogeeta Varadarajalu

**Affiliations:** Division of Infectious Diseases, Department of Medicine, Miller School of Medicine, University of Miami, Miami, FL 33136, USA; yogeetanaidu@gmail.com

**Keywords:** lymphadenitis, *Cryptococcus neoformans*, immune reconstitution, HIV

## Abstract

Cryptococcosis is a fungal infection that is typically associated with acquired immunodeficiency syndrome (AIDS). The advent of highly active antiretroviral therapy has decreased the frequency of this infection, but has led to the emergence of atypical cases of immune reconstitution inflammatory syndrome (IRIS). Here, we describe the case of a 40-year-old man who was diagnosed with HIV infection and cryptococcal meningitis. He was successfully treated with antifungals and then started antiretroviral therapy. The patient returned to the hospital 15 months later complaining of fever, pain, and neck swelling. A computed tomography (CT) scan revealed a conglomerate of necrotic lymph nodes in the supraclavicular region. He underwent biopsy and histology showed granulomatous inflammation with fungal elements, consistent with *Cryptococcus*. He tested positive for serum cryptococcal antigen. The patient was treated with liposomal amphotericin and flucytosine. After induction therapy, he was re-started on fluconazole. The final fungal cultures were negative. We attributed our patient’s clinical presentation to “paradoxical” IRIS, which was associated with his previously treated cryptococcosis. Near resolution of the supraclavicular mass was noted at the 3-month follow-up.

**Figure 1:**
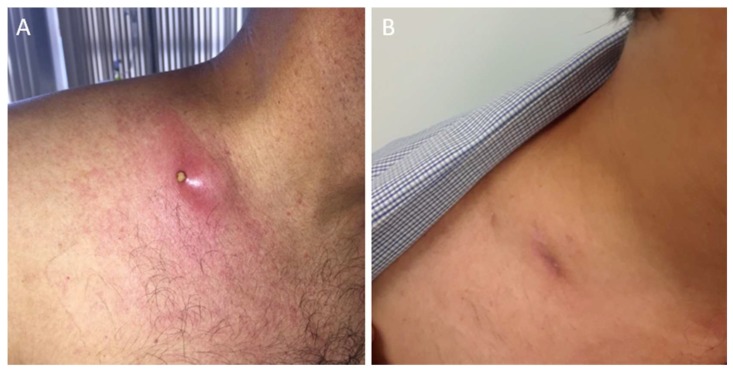

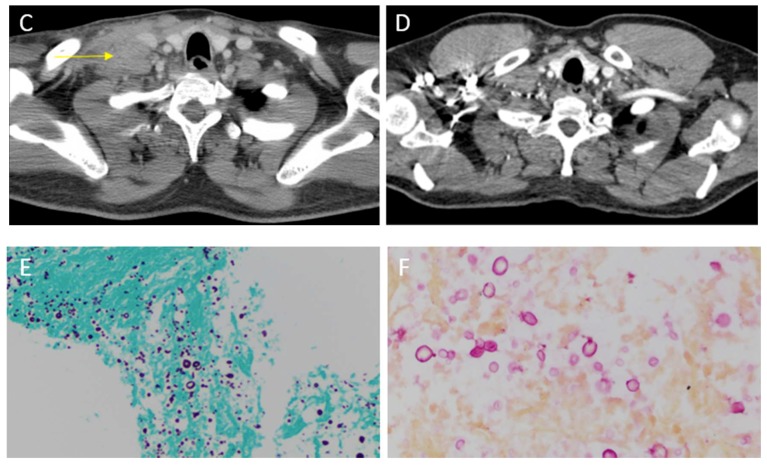
A 40-year-old Hispanic man presented to the hospital with headaches, dizziness, and progressive altered mental status for 1 week. His neurologic exam was significant for lethargy and neck stiffness. No focal deficits were noted. He was found to be infected with HIV. His CD4 count was 40 cells/μL, and his HIV viral load was 400,000 copies/mL. A lumbar puncture was performed and cerebrospinal fluid (CSF) analysis showed 15 white blood cells/mm^3^, a protein content of 60 mg/dL, and a glucose level of 20 mg/dL. The cryptococcal antigen from CSF was 1:512. A fungal culture from CSF isolated *Cryptococcus neoformans.* The patient was treated with 4 mg/kg/day of liposomal amphotericin and 100 mg/kg/day of flucytosine for 2 weeks (induction therapy). He developed marked improvement of his neurologic symptoms. Treatment was followed by consolidation therapy with 400 mg of fluconazole daily for 8 weeks. After 2 weeks of consolidation therapy, the patient started antiretroviral therapy, consisting of tenofovir alafenamide/emtricitabine, dolutegravir, and boosted darunavir (given the presence of the M184V mutation on the HIV genotype). After completing consolidation treatment, the patient was maintained on 200 mg fluconazole daily. He presented 15 months later with a painful neck swelling, fever, and chills. He stated that he had been compliant with HIV therapy and fluconazole. A physical exam revealed an enlarged and tender lymph node with purulent drainage in the supraclavicular region (**A**). To better characterize these findings and to evaluate extension into adjacent lymph nodes, we ordered a cervical computed tomography (CT) scan with contrast. This image showed a 6.2 × 4.3 cm heterogeneous mass (yellow arrow) anterolateral to the right scalene muscles, likely representing a conglomerate of necrotic lymph nodes (**C**). Laboratory studies showed a CD4 count of 227 cells/μL and an undetectable HIV viral load. He tested positive for serum cryptococcal antigen with a titer of 1:20. The patient was started on 15 mg/kg of vancomycin every 12 h and 1 g of ceftriaxone daily for a presumptive bacterial infection, and he continued with 200 mg of fluconazole daily. He underwent an ultrasound (US)-guided core biopsy of the lymph node. Histopathology showed granulomatous inflammation and necrosis with encapsulated pleomorphic round yeast with features suggesting *Cryptococcus*. Grocott’s Methenamine Silver stain highlighted the fungal elements (**E**). Their thick mucinous capsule stained bright red with mucicarmine special stain (**F**). Antibiotics were discontinued and the patient re-started induction therapy with liposomal amphotericin and flucytosine for possible cryptococcal relapse. He completed 2 weeks of induction therapy. After achieving significant clinical improvement, the patient was discharged with 400 mg of fluconazole daily. The final fungal cultures remained negative. At the 3-month follow-up, a physical exam showed near resolution of lymphadenitis (**B**). In addition, a CT scan showed a marked reduction in the necrotic mass (**D**). Cryptococcosis is a fungal infection typically associated with T-cell deficiencies, particularly acquired through immunodeficiency syndrome (AIDS), and it usually manifests as cryptococcal meningitis [1]. Although antiretroviral therapy has intensely reduced AIDS-related opportunistic infections, the restoration of CD4 cells can induce an immune reconstitution inflammatory syndrome (IRIS), characterized by a deregulated inflammatory response to specific infectious or non-infectious antigens. This response may lead to clinical deterioration caused by a previously unrecognized subclinical disease with viable opportunistic pathogens, which is known as “unmasking” IRIS. A similar process resulting from the worsening of a previously well-treated infection can also occur and is called “paradoxical” IRIS. In the latter, the pathogens are no longer viable, and cultures remain negative [2]. A wide extent of infections related to IRIS has been reported, of which cryptococcosis, tuberculosis, and *Mycobacterium avium* complex are the most common [3]. The overall incidence of IRIS associated cryptococcosis varies from 10 to 30%. It commonly presents as recurrent meningitis. Lymphadenitis is considered a rare event with only a few cases reported in the literature [4,5]. We believe our patient developed cryptococcal IRIS manifesting as lymphadenitis. His CD4 count markedly increased from 40 to 227 cells/μL after 15 months of antiretroviral therapy, which precipitated this inflammatory reaction. Our patient’s clinical presentation was consistent with the paradoxical type of IRIS, because it occurred while the patient was on effective antifungal therapy. Furthermore, despite visualizing fungal elements in the histopathology, no viable organisms were obtained from cultures. The treatment of this condition depends on the type of IRIS. For unmasking cryptococcal IRIS, the treatment is standard antifungal therapy. On the other hand, paradoxical cryptococcal IRIS does not require a new course of induction and consolidation therapy, because the pathogens are no longer viable. Many clinicians end up giving standard antifungal therapy while waiting for negative cultures [6], which also occurred in our case. Adjunctive therapy with steroids can be considered in patients with severe paradoxical responses. Discontinuation of antiretrovirals is not recommended [7]. In our patient, anti-inflammatory medications were not needed. A similar case of cervical lymphadenitis by *Cryptococcus* was described by Bhuyan et al. [8]. They achieved a definite diagnosis by fine-needle aspiration of the involved lymph node and subsequent histopathological examination. The characteristic capsule of *Cryptococcus* is used as a diagnostic tool. The hematoxylin-eosin and Nigrosin stains create a negative image, visualizing the thick capsule as a clear halo on a dark background. Other special stains used in the laboratory to visualize the gelatinous capsule are mucicarmine and methenamine silver stains [8]. Our case highlights the importance of considering cryptococcal-related IRIS in the differential diagnosis of lymphadenitis in HIV-infected individuals. We advocate the use of US-guided biopsy of affected lymph nodes and tissue cultures for accurate and prompt diagnosis of this condition. Resolution of symptoms generally occurs with conservative therapy and continuation of antiretrovirals.
